# Comparative assessment of the capability of machine learning-based radiomic models for predicting omental metastasis in locally advanced gastric cancer

**DOI:** 10.1038/s41598-024-66979-x

**Published:** 2024-07-13

**Authors:** Ahao Wu, Lianghua Luo, Qingwen Zeng, Changlei Wu, Xufeng Shu, Pang Huang, Zhonghao Wang, Tengcheng Hu, Zongfeng Feng, Yi Tu, Yanyan Zhu, Yi Cao, Zhengrong Li

**Affiliations:** 1 Department of Digestive Surgery, Digestive Disease Hospital, The First Affiliated Hospital of Nanchang University, Nanchang, 330006 Jiangxi Province China; 2 Department of Gastrointestinal Surgery, The Second Affiliated Hospital, Nanchang University, Nanchang, 330006 Jiangxi Province China; 3 Medical Innovation Center, The First Affiliated Hospital of Nanchang University, Nanchang, China; 4 Department of Pathology, The First Affiliated Hospital of Nanchang University, Nanchang, 330006 Jiangxi Province China; 5 Department of Radiology, The First Affiliated Hospital, Nanchang University, Nanchang, 330006 Jiangxi Province China; 6 Department of Digestive Surgery, Digestive Disease Hospital, The Third Affiliated Hospital of Nanchang University, Nanchang, 330006 Jiangxi Province China; 7 General Surgery Department of Jiangxi Provincial People’s Hospital, Nanchang, 330006 Jiangxi Province China

**Keywords:** Machine learning, Omental metastases, Radiomics, Locally advanced gastric cancer, Positive predictive value, Cancer, Cancer models, Gastrointestinal cancer

## Abstract

The study aims to investigate the predictive capability of machine learning algorithms for omental metastasis in locally advanced gastric cancer (LAGC) and to compare the performance metrics of various machine learning predictive models. A retrospective collection of 478 pathologically confirmed LAGC patients was undertaken, encompassing both clinical features and arterial phase computed tomography images. Radiomic features were extracted using 3D Slicer software. Clinical and radiomic features were further filtered through lasso regression. Selected clinical and radiomic features were used to construct omental metastasis predictive models using support vector machine (SVM), decision tree (DT), random forest (RF), K-nearest neighbors (KNN), and logistic regression (LR). The models’ performance metrics included accuracy, area under the curve (AUC) of the receiver operating characteristic curve, sensitivity, specificity, positive predictive value (PPV), and negative predictive value (NPV). In the training cohort, the RF predictive model surpassed LR, SVM, DT, and KNN in terms of accuracy, AUC, sensitivity, specificity, PPV, and NPV. Compared to the other four predictive models, the RF model significantly improved PPV. In the test cohort, all five machine learning predictive models exhibited lower PPVs. The DT model demonstrated the most significant variation in performance metrics relative to the other models, with a sensitivity of 0.231 and specificity of 0.990. The LR-based predictive model had the lowest PPV at 0.210, compared to the other four models. In the external validation cohort, the performance metrics of the predictive models were generally consistent with those in the test cohort. The LR-based model for predicting omental metastasis exhibited a lower PPV. Among the machine learning algorithms, the RF predictive model demonstrated higher accuracy and improved PPV relative to LR, SVM, KNN, and DT models.

## Introduction

Gastric cancer is one of the most common malignant tumors worldwide. The incidence and mortality rates of gastric cancer rank fifth and fourth globally, and in China, they rank third^1^. In recent years, treatment options for gastric cancer have expanded significantly^2–6^, including surgery, chemotherapy, targeted therapy and immunotherapy^7–11^; but radical surgery remains the primary treatment modality for locally advanced gastric cancer (LAGC)^12,13^. For LAGC patients without omental metastasis, radical gastrectomy with omentum preservation offers advantages such as shorter operating time, less intraoperative blood loss, and fewer postoperative complications^14,15^. Therefore, accurate preoperative staging of gastric cancer patients is of great importance for individualized treatment.

Radiomics has been increasingly applied in gastric cancer research in recent years. Its principle involves converting medical images into numerical variables for statistical analysis^16^. Radiomics plays a crucial role in predicting gastric cancer lymph node metastasis, N staging, neoadjuvant chemotherapy efficacy, postoperative local recurrence, and long-term survival^17–19^. Additionally, at the molecular level, radiomics has been employed to investigate immune cell infiltration and assess the sensitivity of immune therapy^20^. The application of radiomics has improved preoperative staging for gastric cancer patients, contributing significantly to individualized treatment. However, even when using the same radiomic techniques, the accuracy of predictions varies across studies due to the diverse predictive methods employed by researchers. For example, in predicting gastric cancer lymph node metastasis using computed tomography (CT) radiomic features, the use of different predictive models can result in substantial variations in prediction accuracy^17,21^. Consequently, there is a need to further evaluate the accuracy among various predictive models to identify the optimal model.

With the development of artificial intelligence, machine learning algorithms have been progressively developed and widely applied in the field of gastric cancer. Examples include constructing gastric cancer survival and neoadjuvant chemotherapy benefit prediction models using support vector machine (SVM)^22^; predicting early gastric cancer patients’ lymph node metastasis status using decision tree (DT) models^23^; predicting Chinese gastric cancer patients’ HER2 status using random forest (RF) models^24^; constructing artificial neural network models for screening and diagnosing non-invasive gastric cancer^25^; and applying K-nearest neighbors (KNN) algorithms to classify gastric cancer lymph node metastasis^26^. Although the development of machine learning algorithms has significantly enhanced the accuracy of predictive models in tumor diagnosis, adjuvant therapy benefits, and survival prognosis, the predictive abilities of different machine learning models for the same diagnostic prediction are inconsistent^27^. The selection of a suitable machine learning prediction model requires further research.

Our previous study on constructing LAGC omental metastasis prediction models demonstrated that although the predictive model for omental metastasis constructed using logistic regression (LR) had relatively high accuracy and area under the curve (AUC), the positive predictive value (PPV) was less than satisfactory^28^. This trend was particularly evident in unbalanced binary classification studies^27–29^. Therefore, the objective of this study is to further investigate the predictive capabilities of machine learning algorithms in LAGC omental metastasis and compare the performance of various machine learning predictive models.

## Materials and methods

### Patients and study design

**Figure 1:**
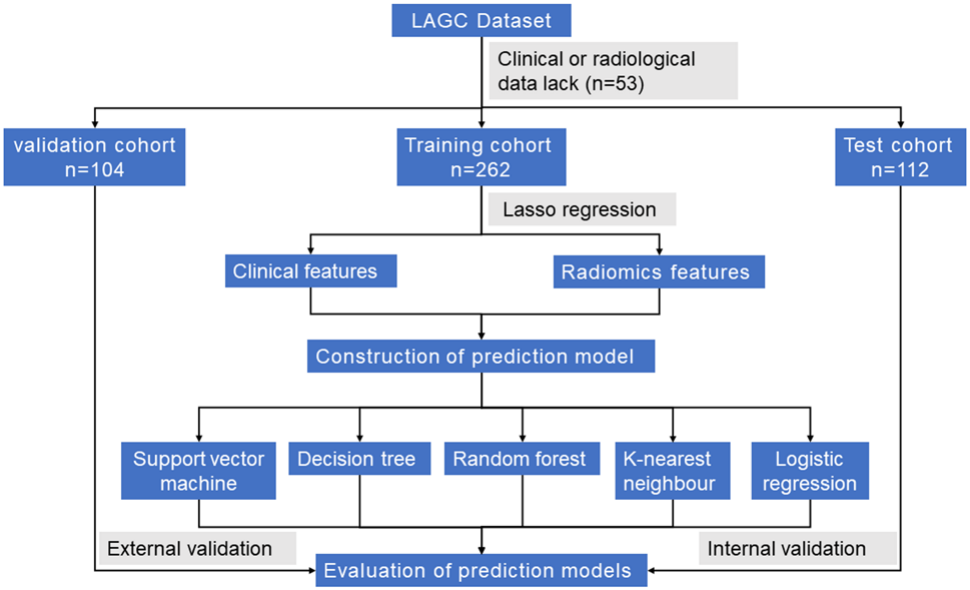
Flow of the study design.

A retrospective collection of 478 pathologically confirmed LAGC patients was conducted from April 2019 to July 2022 at the First Affiliated Hospital of Nanchang University and the Second Affiliated Hospital of Nanchang University, including clinical features and arterial phase CT images. Of these, 374 patients from the First Affiliated Hospital of Nanchang University were randomly divided into a training cohort and a test cohort at a 7:3 ratio. Additionally, 104 patients from the Second Affiliated Hospital of Nanchang University were used as a validation cohort. Inclusion criteria were as follows: (1) enhanced abdominal CT examination within 2 weeks before surgery; (2) LAGC patients underwent radical gastrectomy with omentectomy; (3) histologically confirmed primary gastric adenocarcinoma; (4) postoperative pathology confirmed as T3/T4 stage. Exclusion criteria were: (1) patients with incomplete clinical data; (2) postoperative pathology confirmed as T1/T2 stage; (3) CT images with artifacts or inadequate inflation, insufficient image quality for diagnosis; (4) neoadjuvant chemoradiotherapy before surgery. The study design flowchart is shown in Fig. 1. The Medical Research Ethics Board of the First Affiliated Hospital of Nanchang University, China, waived the informed consent in the main manuscript (The Ethics Board approval number: (2022)CDYFYYLK(08-007)). All methods were performed in accordance with the relevant guidelines and regulations.

### Radiomic feature extraction and selection

Arterial phase CT images were downloaded from the imaging center of the First Affiliated Hospital and the Second Affiliated Hospital of Nanchang University and saved in DICOM format. Tumor segmentation was performed manually by two radiologists with more than 5 years of experience in abdominal radiology using 3D Slicer software (version 4.11). The segmented image files were saved in Nrrd format. Pyradiomics, a plugin in 3D Slicer, was used to extract features from all patients’ Nrrd format files. Feature selection was performed using the least absolute shrinkage and selection operator (LASSO) regression method in R software (version 4.13). The “glmnet” package was downloaded in R software. To construct the optimal LASSO regression model, we generated 200 lambda values ranging from 0 to 0.05. The lambda value corresponding to the minimum mean square error of the model is the optimal lambda. The selected features can be obtained by inputting the optimal lambda into the glmnet function.

### SVM

Support vector machine (SVM) is a powerful classifier. Its principle is to create a decision boundary between two classes and then predict labels using one or more feature vectors. This decision boundary is often a hyperplane whose direction is as far away as possible from the data points closest to each class. These closest points are called support vectors. The “e1071” package was downloaded in R language. The SVM model performed hyperparameter optimization to determine the optimal cost and gamma values and construct the best SVM model.

### DT and RF

We used the “rpart” package in R language for decision analysis. DT classification proceeds from the root to the leaves, using statistical measures such as the Gini index and gain ratio to describe the purity and determine the optimal split. When there is only one class of samples in the child node, the Gini index is 0, and the child node stops splitting. We first constructed an original decision tree using all the independent variables in the training cohort and then built the optimal DT based on the best node for tree post-pruning. RF is a collection of DTs that individually serve as weak classifiers but together form a robust prediction. This method randomly extracts bootstrap samples and features to build each classification and regression tree. Hyperparameters in the study included the number of trees and leaf nodes in the forest. The optimal model was selected by hyperparameter optimization.

### KNN

The “knn” package was downloaded in R language, and the LAGC omental metastasis diagnostic model was constructed using the training cohort data. If the test sample is closest in feature space to k determined samples, it is considered to belong to the same class as these k samples. Too large or too small k values affect the accuracy of classification. Therefore, we performed hyperparameter optimization by setting the k-value range from 0 to 100, to construct the optimal model.

### LR

The “rms” package was downloaded in R language. Stepwise regression using forward, backward, and forward–backward methods was applied to construct the LR diagnostic model in the training cohort. The best diagnostic model was selected based on the Akaike information criterion (AIC) size. To make the evaluated models more accurate and reliable, all diagnostic models underwent four-fold cross-validation. The training cohort data were divided into four parts, with one part used as the validation set. After four tests, changing the validation set each time, the average of the four model results was taken as the final result. On the other hand, the test cohort served as internal data to evaluate the reliability of the model, and an external validation cohort was used to evaluate the model. Model evaluation metrics included area under the curve (AUC) of the receiver operating characteristic (ROC) curve, accuracy, sensitivity, specificity, PPV, and negative predictive value (NPV).

### Statistical analysis

Statistical analysis was performed using SPSS software (version 26.0) and R software (version 4.13). Count data were expressed as frequencies, and differences between count data were compared using the chi-square test. Normally distributed measurement data were expressed as mean ± SD and compared using the *t*-test for differences between normally distributed variables. Non-parametric tests were used for differences analysis of non-normal distribution and heterogeneous variance measurement data. Differences between multiple groups were compared using one-way analysis of variance. A p-value < 0.05 was considered statistically significant.

### Ethics approval and informed consent

This study was approved by the Ethics Committee of the First Affiliated Hospital of Nanchang University [Ethics Committee Approval No.: (2022)CDYFYYLK(08-007)]. As this study was retrospective, the Ethics Committee of the First Affiliated Hospital of Nanchang University waived the informed consent for this study. All methods were performed according to relevant guidelines and regulations.

## Results

### Clinical features

Among the 478 eligible patients, 419 patients had no omental metastasis, and 59 patients had omental metastasis, with an occurrence rate of 14.1%. The training cohort consisted of 262 patients: 189 males and 79 females; 229 had no omental metastasis, and 33 had omental metastases. The test cohort consisted of 112 individuals, 83 males, and 29 females; 13 of them had omental metastases, and 99 did not. The validation cohort consisted of 104 patients, 75 males, and 29 females; 13 patients had omental metastases, and the remaining 91 patients did not. The clinical features of the training, test, and validation cohorts showed no significant statistical differences (Table 1), ensuring the reliability of the results obtained from the test and validation cohorts.

**Table 1:** LAGC patient characteristics in the training, test, and validation cohorts.

Characteristics	Training cohort (n = 262)	Test cohort (n = 112)	Validation cohort (n = 104)	P value
Age(years)	61.78 ± 10.27	62.29 ± 11.68	62.36 ± 11.93	0.867
BMI(Kg/m^2^)	22.14 ± 3.07	22.15 ± 2.89	22.16 ± 4.08	0.998
NLR	3.09 ± 3.04	3.09 ± 3.85	2.97 ± 3.80	0.951
PLR	180.86 ± 102.12	177.26 ± 81.72	164.36 ± 76.98	0.306
Albumin(g/L)	39.02 ± 4.52	39.31 ± 5.30	39.26 ± 5.44	0.844
Tumor size(cm^2^)	21.83 ± 24.73	24.15 ± 24.56	25.10 ± 24.27	0.452
Omental metastases				
No	229	99	91	0.964
Yes	33	13	13	
Gender				
Male	183	83	75	0.692
Female	79	29	29	
CT-reported LN status				
LN ( −)	124	46	46	0.525
LN ( +)	138	66	58	
CEA				
Normal	214	92	84	0.965
Abnormal	48	20	20	
CA125				
Normal	244	108	95	0.295
Abnormal	18	4	9	
CA19-9				
Normal	191	90	84	0.146
Abnormal	71	22	20	
Borrmann classification				
I	7	4	4	0.634
II	77	39	39	
III	168	63	56	
IV	10	6	5	
Tumor location				
Proximal third	34	18	17	0.537
Middle third	70	25	25	
Distal third	155	65	58	
Complete stomach	3	4	4	
Clinical T stage				
cT3	61	27	26	0.717
cT4a	79	26	28	
cT4b	122	59	50	
Clinical N stage				
N0	61	22	21	0.255
N1	39	25	21	
N2	48	24	27	
N3a	67	19	22	
N3b	47	22	13	

*NLR* meutrophil-to-lymphocyte ratio, *PLR* platelet-to-lymphocyte ratio, *BMI* body mass index, *CT* computed tomography, *LN ( −)* lymph note metastasis negative, *LN(* +*)* lymphnode metastasis positive, *CEA* carcinoembryonic antigen, *CA19-9* carbohydrate antigen 19-9, *CA125* cancer antigen 125.

### Radiomic feature and clinical feature selection

**Figure 2:**
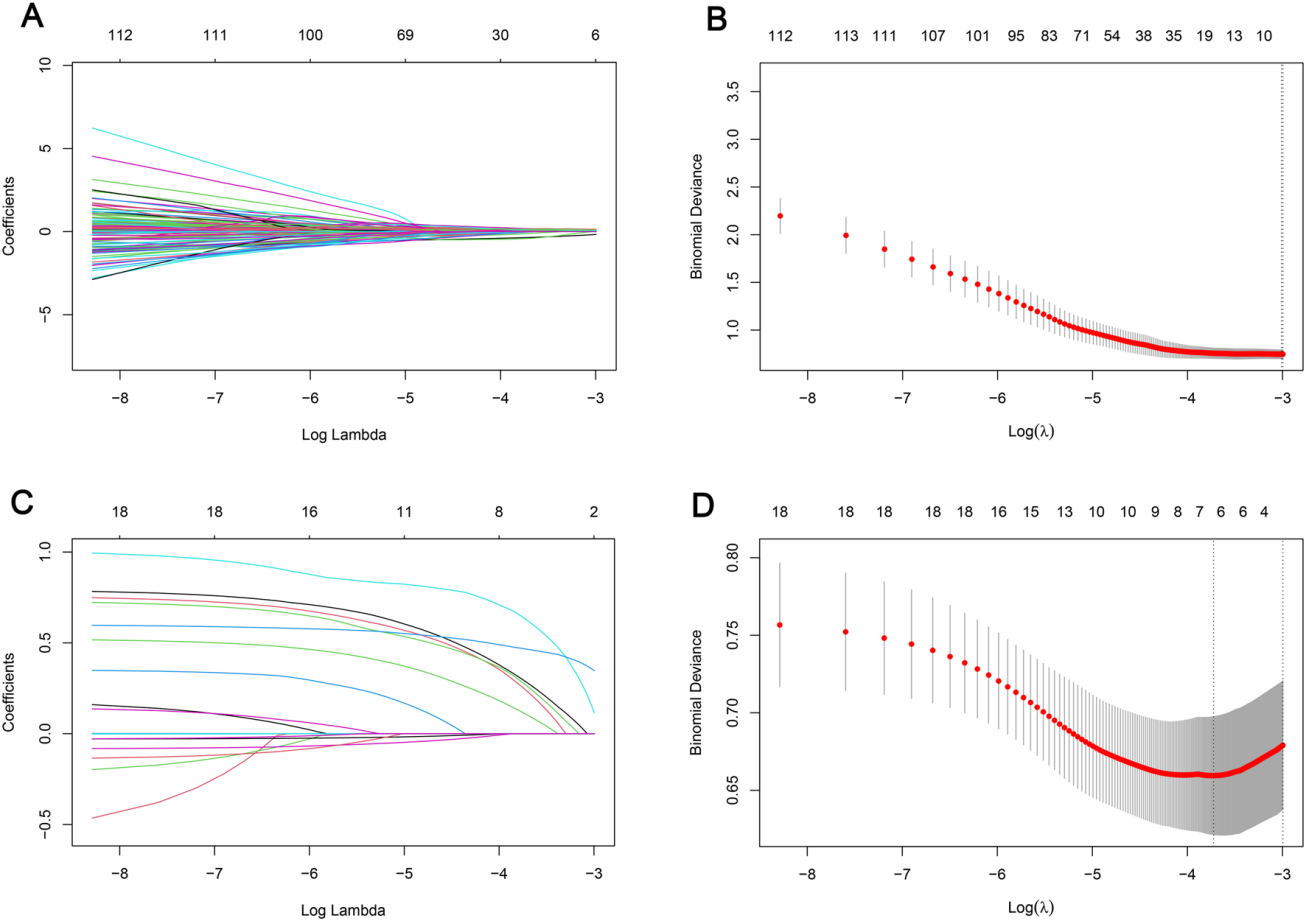
Screening of radiomic feature and clinical feature. (**A**) Relationship between the lambda values and radiomic feature coefficients; (**B**) relationship between the lambda values and bias percentage of radiomic features; (**C**) relationship between the lambda values and clinical feature coefficients; (**D**) relationship between the lambda values and bias percentage of clinical features.

We extracted 864 radiomic features from the arterial phase CT images. Detailed radiomic features are shown in Additional File 1. Features with an intra-class correlation coefficient (ICC) greater than 0.75 were considered stable features, and 548 radiomic features were ultimately selected. The ICC values for radiomic features are detailed in Additional File 2. The radiomic features of the training cohort were analyzed using LASSO regression. As the lambda value increased, the absolute value of the feature coefficients gradually decreased and eventually approached 0 (Fig. 2A). As the lambda value increased, the bias percentage first gradually decreased and then gradually increased. The optimal lambda value corresponds to the minimum bias percentage (Fig. 2B). The selected radiomic features based on the optimal lambda value were diagnostics image original mean (DIOM), original shape maximum 2D diameter slice (OSMDS), original shape maximum 3D diameter (OSMD), original first order kurtosis (OFK), wavelet LH first order kurtosis (WLFK), and wavelet HLH Gldm large dependence high gray level emphasis (WHGL). Similarly, the clinical features of the training cohort were subjected to LASSO regression to select distinct clinical features. The clinical features selected based on the optimal lambda value were CA125 and clinical N staging (Fig. 2C, D).

### SVM-based predictive model

**Figure 3:**
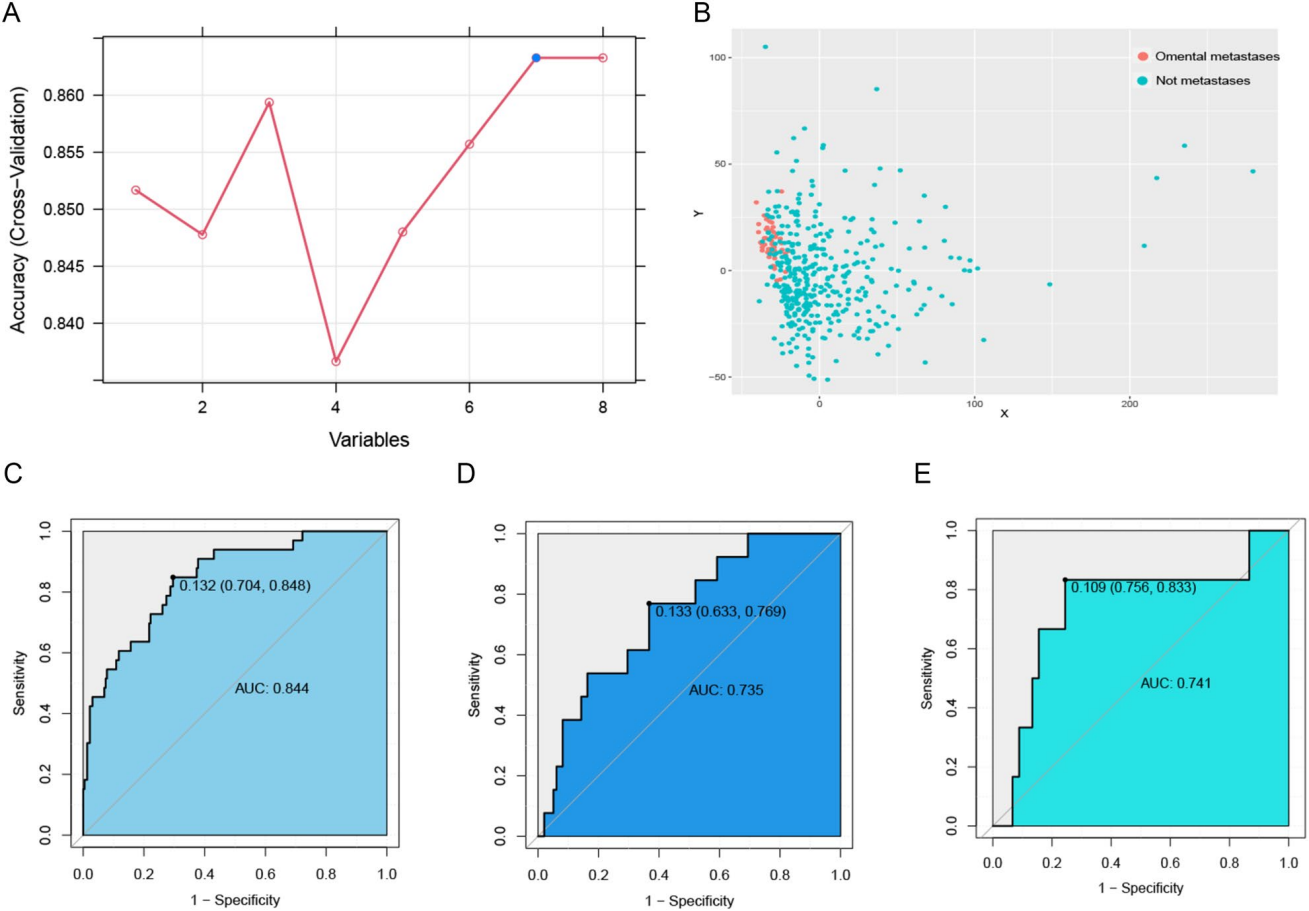
SVM-based capability assessment of the predictive model. (**A**) Number of vectors and model accuracy of the SVM; (**B**) distribution of omental metastasis and non-omental metastasis patients in the two-dimensional space; (**C**) the ROC curve of the training cohort; (**D**) the ROC curve of test cohort; (**E**) the ROC curve of the validation cohort.

In the omental metastasis prediction model constructed using SVM, we determined that the model had the highest accuracy when the vector number was 7 (Fig. 3A). The multidimensional data of LAGC patients were converted into two-dimensional data, and omental metastasis and non-omental metastasis groups had a more distinct concentrated distribution in the two-dimensional space (Fig. 3B). In the training cohort, the AUC value of the predictive model was 0.844; sensitivity and specificity were 0.849 and 0.704, respectively; PPV and NPV were 0.292 and 0.790, respectively (Fig. 3C). In the test cohort, the AUC of the predictive model was 0.735; sensitivity and specificity were 0.769 and 0.622, respectively; PPV and NPV were 0.212 and 0.953, respectively (Fig. 3D). In the validation cohort, the AUC of the predictive model was 0.741; sensitivity and specificity were 0.833 and 0.756, respectively; PPV and NPV were 0.385 and 0.974, respectively (Fig. 3E).

### DT-based predictive model

**Figure 4:**
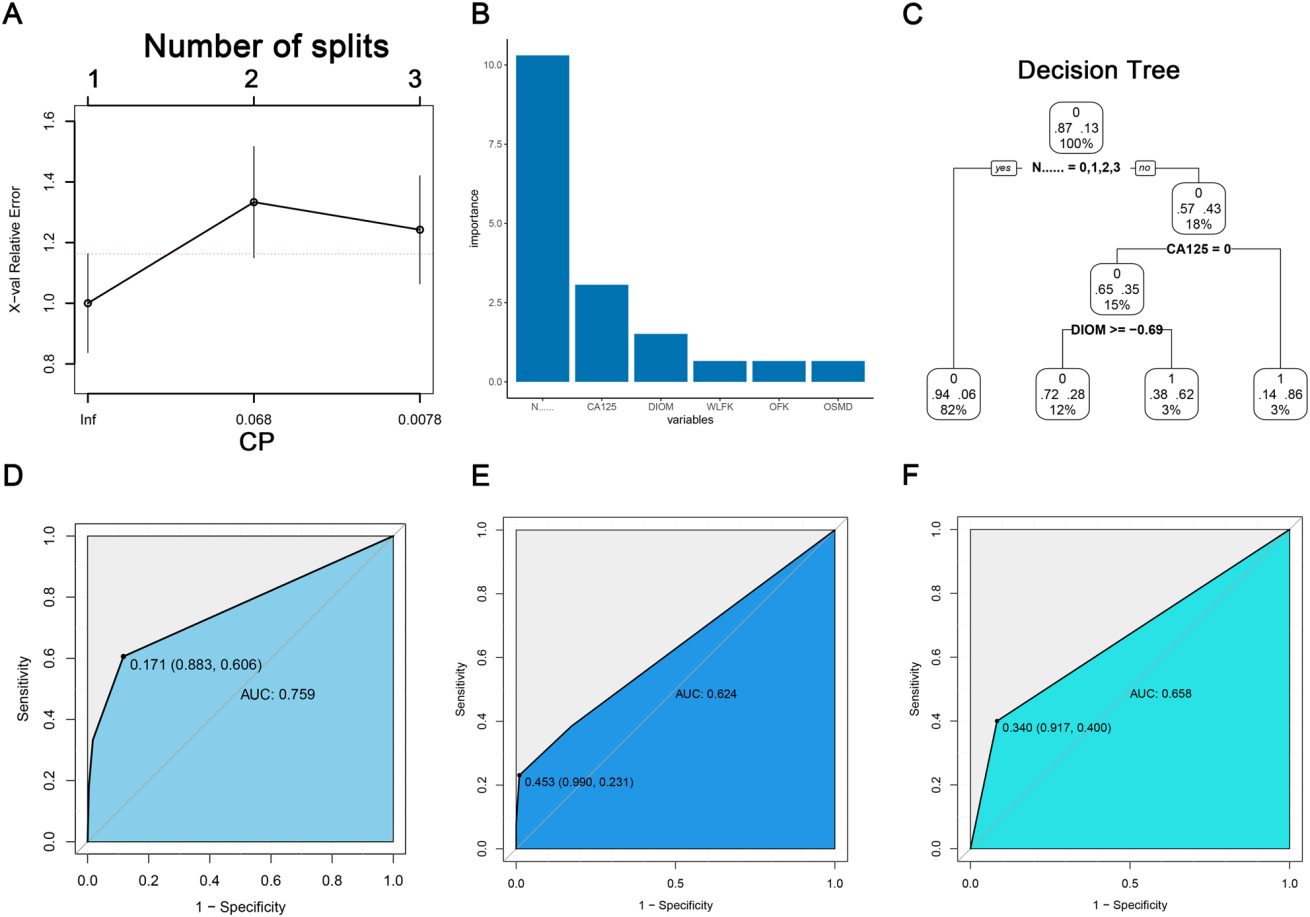
DT-based capability assessment of the predictive model. (**A**) Relationship between the number of splitting points of DT and complexity parameters; (**B**) the significance of clinical features and radiomic features for DT-based predictive model; (**C**) decision tree diagram; (**D**) the ROC curve of the training cohort; (**E**) the ROC curve of the test cohort; (**F**) the ROC curve of the validation cohort.

In DT, we determined the number of tree split nodes to be 3 (Fig. 4A). The variable features of the constructed prediction model were ranked according to their importance, with the top six features being clinical N staging, CA125, DIOM, WLFK, OFK, and OSMD, respectively (Fig. 4B). Based on the DT split node number of 3, we selected the top three important features to construct the DT prediction model (Fig. 4C). In the training cohort, the AUC of the predictive model was 0.759; sensitivity and specificity were 0.606 and 0.883, respectively; PPV and NPV were 0.426 and 0.940, respectively (Fig. 4D). In the test cohort, the AUC of the predictive model was 0.624; sensitivity and specificity were 0.231 and 0.990, respectively; PPV and NPV were 0.227 and 0.910, respectively (Fig. 4E). In the validation cohort, the AUC of the predictive model was 0.658; sensitivity and specificity were 0.400 and 0.917, respectively; PPV and NPV were 0.400 and 0.917, respectively (Fig. 4F).

### RF-based predictive model

**Figure 5:**
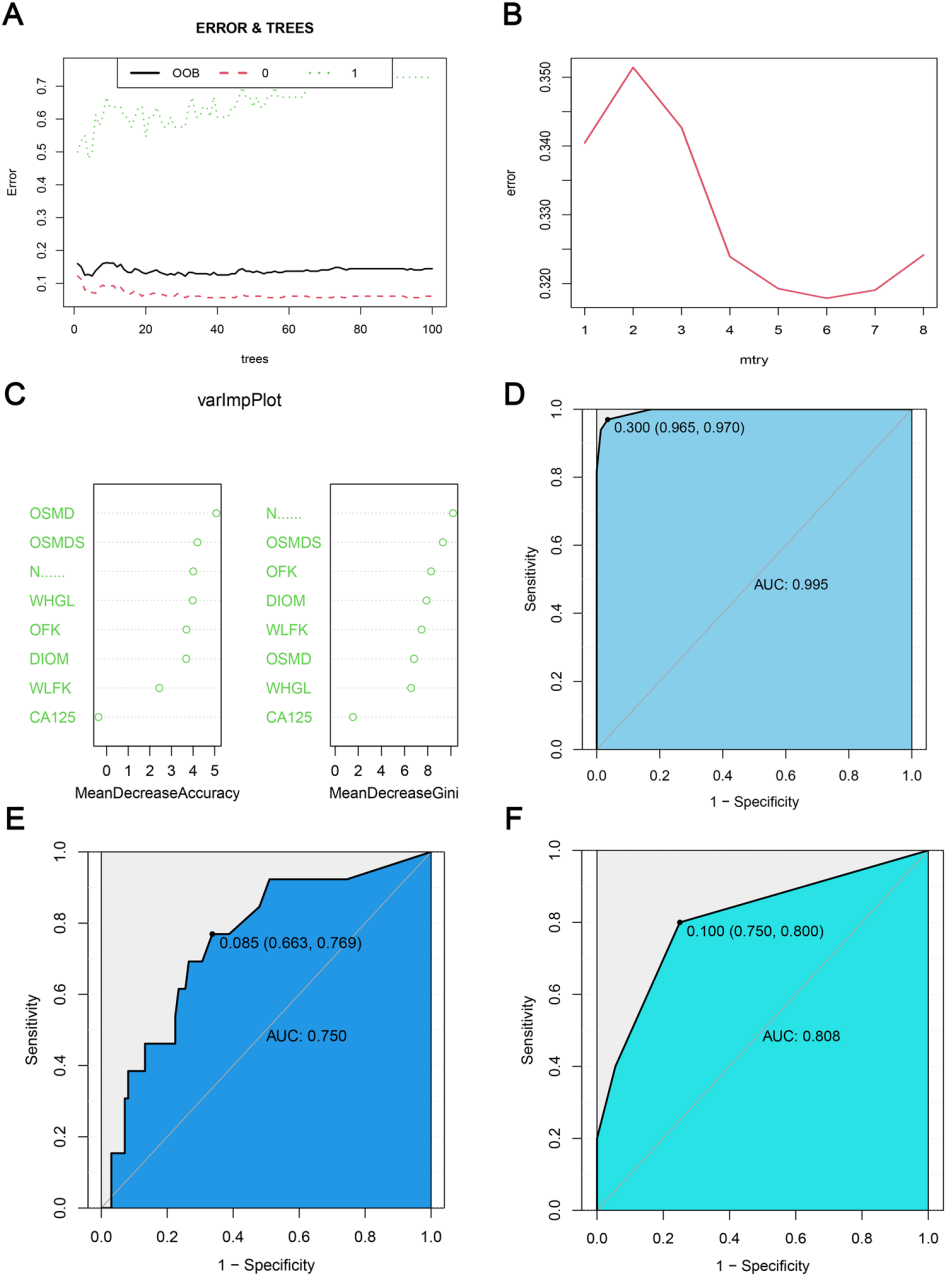
RF-based capability assessment of the predictive model. (**A**) Relationship between the number of trees and OOB error in RF, “0” represents the omental metastasis group, and “1” represents the non-omental metastases group; (**B**) relationship between the number of split points and error in the tree; (**C**) importance of radiomic and clinical features in improving the model accuracy and reducing the Gini coefficient; (**D**) the ROC curve of the training cohort; (**E**) the ROC curve of the test cohort; (**F**) the ROC curve of the validation cohort.

In the RF predictive model, when the number of trees in the model is 5, the out-of-bag (OOB) error reaches the minimum value of 0.122 (Fig. 5A). We then determined the tree split nodes, and when the number of split nodes is 6, the error reaches the minimum value of 0.318 (Fig. 5B). Additionally, we conducted feature importance analysis, and among the eight selected features, OSMD, OSMDS, and N staging played important roles in prediction accuracy. N staging, OSMDS, and OFK played important roles in reducing the Gini coefficient of the predictive model (Fig. 5C). In the training cohort, the AUC value of the predictive model was 0.995, sensitivity and specificity were 0.970 and 0.965, respectively; PPV and NPV were 0.800 and 0.995, respectively (Fig. 5D). In the test cohort, the AUC value of the predictive model was 0.750, sensitivity and specificity were 0.769 and 0.663, respectively; PPV and NPV were 0.233 and 0.956, respectively (Fig. 5E). In the validation cohort, the AUC value of the predictive model was 0.808, sensitivity and specificity were 0.750 and 0.800, respectively; PPV and NPV were 0.308 and 0.964, respectively (Fig. 5F).

### KNN-based predictive model

**Figure 6:**
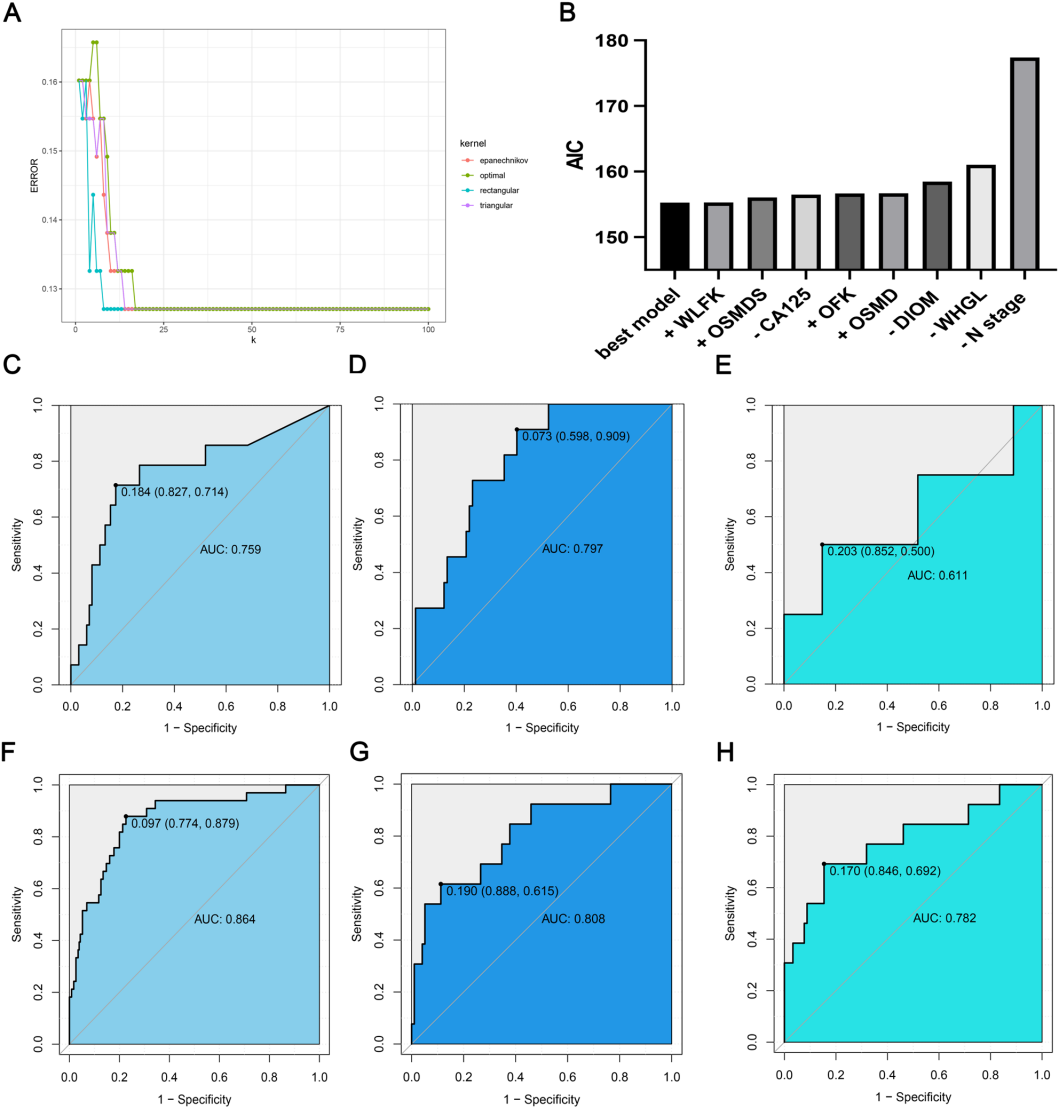
KNN and LR-based capability assessment of the predictive models. (**A**) Screening for the best kernel function and k-value; (**B**) best predictive model and each feature’s AIC value; (**C**) the ROC curve of the training cohort in the LR-based predictive model; (**D**) the ROC curve of the test cohort in the LR-based predictive model; (**E**) the ROC curve of the validation cohort in the LR-based predictive model; (**F**) the ROC curve of the training cohort in the KNN-based predictive model; (**G**) the ROC curve of the test cohort in the KNN-based predictive model; (**H**) the ROC curve of the validation cohort in the KNN-based predictive model.

Through the hyperparameter optimization of the KNN function, we found that the best kernel function for the predictive model was “triangular,” and the best k-value was 14 (Fig. 6A). We constructed the predictive model based on these conditions. In the training cohort, the AUC value of the predictive model was 0.759, sensitivity and specificity were 0.714 and 0.827, respectively; PPV and NPV were 0.370 and 0.952, respectively (Fig. 6C). In the test cohort, the AUC value of the predictive model was 0.797, sensitivity and specificity were 0.909 and 0.598, respectively; PPV and NPV were 0.227 and 0.980, respectively (Fig. 6D). In the validation cohort, the AUC value of the predictive model was 0.611, sensitivity and specificity were 0.500 and 0.852, respectively; PPV and NPV were 0.333 and 0.920, respectively (Fig. 6E).

### LR-based predictive model

Through LR analysis, we found that when the predictive model consists of N staging, CA125, DIOM, and WHGL, the regression model has an optimal fitting state, with an AIC value of 155.24. When the predictive model removes these features or adds other features, the AIC value will increase (Fig. 6B). In the training cohort, the AUC value of the regression predictive model was 0.864, sensitivity and specificity were 0.879 and 0.774, respectively; PPV and NPV were 0.358 and 0.978, respectively (Fig. 6F). In the test cohort, the AUC value of the predictive model was 0.808, sensitivity and specificity were 0.615 and 0.888, respectively; PPV and NPV were 0.210 and 0.941, respectively (Fig. 6G). In the validation cohort, the AUC value of the predictive model was 0.782, sensitivity and specificity were 0.692 and 0.846, respectively; PPV and NPV were 0.250 and 0.953, respectively (Fig. 6H).

### Comparison of predictive abilities of various models

**Figure 7:**
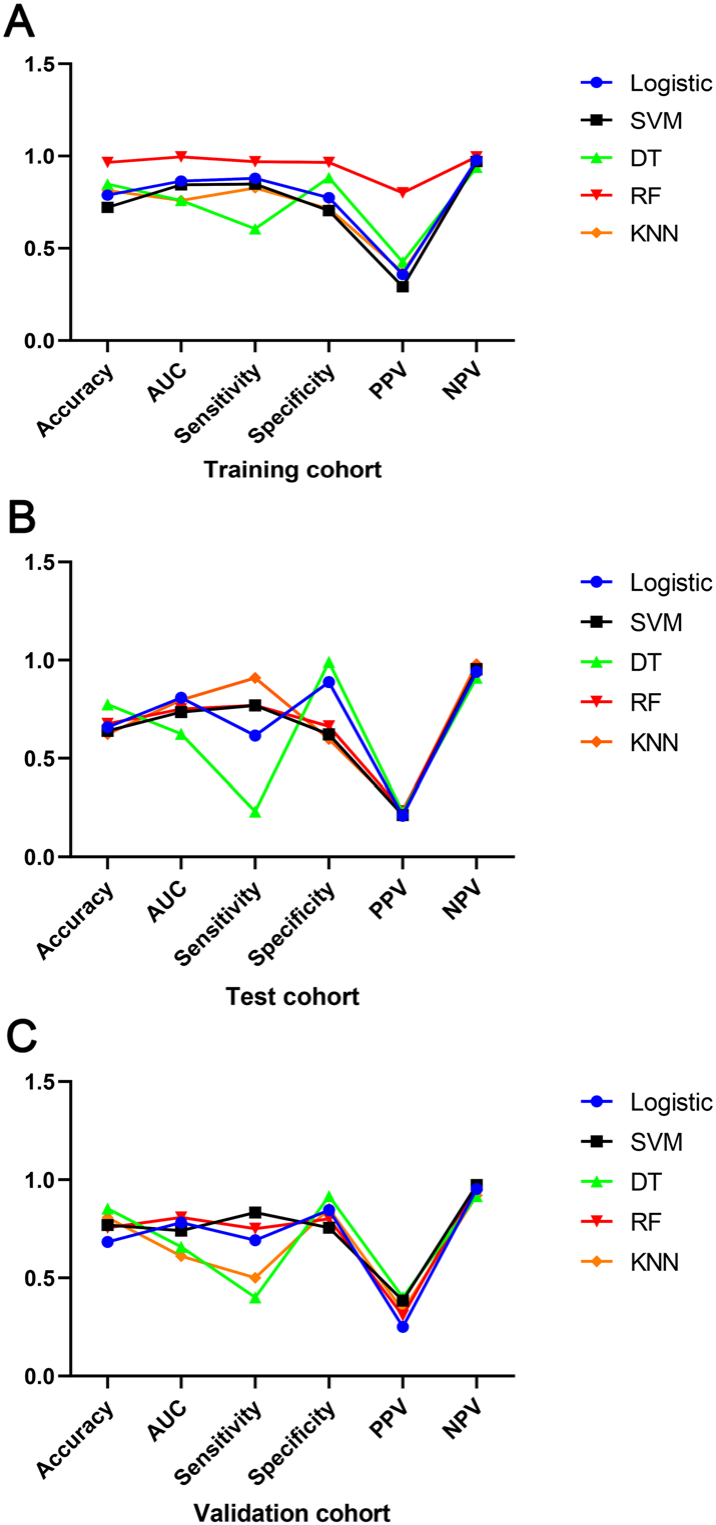
Line graphs depicting the predictive power of machine learning-based models. (**A**) The training cohort; (**B**) the test cohort; (**C**) the validation cohort.

The predictive abilities of omental metastasis models in LAGC constructed using various machine learning methods are shown in Table 2. In the training cohort (Fig. 7A), the RF predictive model had better accuracy, AUC, sensitivity, specificity, PPV, and NPV compared to LR, SVM, DT, and KNN. The RF predictive model achieved a significant improvement in PPV compared to the other four predictive models. The DT predictive model had a lower sensitivity compared to the other four predictive models, with a sensitivity of only 0.606. In the test cohort (Fig. 7B), all five machine learning-constructed predictive models had a relatively low PPV. The evaluation indicators of the DT predictive model were more significantly different compared to the other four predictive models, with a sensitivity of 0.231 and specificity of 0.990 for the DT predictive model. The LR-constructed predictive model had the lowest PPV of 0.210 compared to the other four predictive models. In the external validation cohort (Fig. 7C), the evaluation indicator results of the predictive models were generally similar to those in the test cohort.

**Table 2:** A comparison of omental metastatic models’ prediction ability.

Characteristics	Logistic	SVM	DT	RF	KNN
Training cohort					
Accuracy	0.788	0.722	0.848	0.966	0.813
AUC	0.864	0.844	0.759	0.995	0.759
Sensitivity	0.879	0.849	0.606	0.970	0.827
Specificity	0.774	0.704	0.883	0.965	0.714
PPV	0.358	0.292	0.426	0.800	0.370
NPV	0.978	0.970	0.940	0.995	0.953
Test cohort					
Accuracy	0.658	0.640	0.775	0.676	0.624
AUC	0.808	0.735	0.624	0.750	0.797
Sensitivity	0.615	0.770	0.231	0.769	0.909
Specificity	0.888	0.622	0.990	0.663	0.598
PPV	0.209	0.213	0.227	0.233	0.227
NPV	0.941	0.953	0.910	0.956	0.980
Validation cohort					
Accuracy	0.683	0.769	0.854	0.756	0.807
AUC	0.782	0.741	0.658	0.808	0.611
Sensitivity	0.692	0.833	0.400	0.750	0.500
Specificity	0.846	0.756	0.917	0.800	0.852
PPV	0.250	0.385	0.400	0.308	0.333
NPV	0.953	0.974	0.917	0.964	0.920

## Discussion

Previous prospective OMEGA studies have shown that radical surgery for LAGC can preserve omentum without tumor metastasis^30^. A phase II randomized controlled study demonstrated that omentum-preserving radical surgery for advanced gastric cancer has advantages such as shorter operation time and less bleeding^31^. After propensity score matching, RI et al.^32^ collected 263 cases of advanced gastric cancer radical surgery patients in each group, and there were no significant differences in overall survival and tumor recurrence between the omentum preservation group and the omentum resection group. Therefore, precise preoperative assessment of omental metastasis in advanced gastric cancer is of great importance for the selection of surgical methods. Our previous study constructed an LR predictive model for LAGC omental metastasis based on radiomic features and clinical features^28^. The LR predictive model had an AUC of 0.871, sensitivity of 0.828, specificity of 0.864, PPV of 0.455, NPV of 0.974, and accuracy of 0.860. The inclusion of radiomic features significantly improved the accuracy of the omental metastasis prediction model. Although our LR predictive model had relatively high accuracy, the PPV was only 0.455, meaning that half of the patients we predicted to have omental metastasis were false positives. To this end, we further explored the predictive models of omental metastasis constructed by LR, SVM, DT, RF, and KNN machine learning methods and compared their predictive performance.

Our study results showed that in the training cohort, the overall performance indicators of the RF predictive model were superior to LR, SVM, DT, and KNN. In particular, the PPV of the RF predictive model showed significant improvement compared to the LR predictive model. Although the improvement in PPV for the RF predictive model in the test and validation cohorts was not as substantial as in the training cohort, the PPV of the RF predictive model was still better than that of the LR predictive model. In this study, the PPV of the LR predictive model was 0.358. In other binary classification studies with unbalanced samples, the LR predictive model also had a low PPV. In the prediction study of pathological complete response (PCR) after neoadjuvant chemoradiotherapy in locally advanced rectal cancer (LARC) patients, the PPV of the LR predictive model constructed by Huh et al. was only 0.255^33^. In a study predicting PCR of rectal cancer using magnetic resonance imaging radiomic features, the performance indicators of the RF predictive model were significantly better than those of SVM, KNN, and LR predictive models^34^. In their study, the PPV of the RF predictive model was 0.759, and that of the LR predictive model was 0.354. The better performance of the RF predictive model compared to the LR predictive model may be because traditional risk prediction models are based on regression models, such as LASSO regression and logistic regression. These methods have certain limitations, as they cannot analyze nonlinear relationships and are susceptible to variable selection and confounding factors^35,36^. Machine learning methods provide algorithms for understanding patterns in large, complex, and heterogeneous data. Recursive partitioning, especially random forests, can handle a large number of predictive variables even in the presence of complex interactions^37,38^.

Our research also found that not all machine learning predictive models have good performance in predicting omental metastasis in LAGC. While the DT predictive model demonstrated relatively high accuracy in the training, test, and validation cohorts, its sensitivity was the worst compared to LR, SVM, RF, and KNN predictive models. In the training cohort, the sensitivity of the DT predictive model was 0.606, while in the test cohort, it was 0.231, and in the validation cohort, it was 0.400. Although the DT predictive model had high accuracy, the misdiagnosis rate of omental metastasis-positive patients in the test and validation cohorts was greater than 50%. DT analysis has been proven to be useful in the clinical treatment decision-making process for disease diagnosis and survival prediction^39,40^. In many DT predictive model studies, researchers only evaluated the AUC value and accuracy of the model, without further analysis of sensitivity and specificity. The reason for the low sensitivity of the DT predictive model in this study may be due to the unbalanced distribution of binary classification samples, with omental metastasis patients accounting for only 14.1% of all patients. Furthermore, the DT model is a simple and easy-to-use non-parametric classifier that does not consider the interaction between variables during the tree formation process and only considers single variables for each screening^41^.

The overall performance metrics of the SVM predictive model were slightly lower than those of the LR predictive model in the training cohort. In the test and validation cohorts, the evaluation metrics of the SVM and LR predictive models differed, with the LR predictive model having higher AUC and specificity than the SVM predictive model, while the SVM predictive model had higher sensitivity and PPV than the LR predictive model. The performance metrics of the KNN predictive model were generally similar to those of the LR predictive model in the training and test cohorts. However, in the validation cohort, the KNN predictive model had poorer AUC and sensitivity than the LR predictive model, with an AUC value of 0.611 and a sensitivity of 0.500. In Huang et al.’s^27^ study using clinical features to construct LR models for predicting PCR in locally advanced rectal cancer, the PPV of the LR predictive model was 0.390, significantly lower than KNN, SVM, and other machine learning predictive models. In contrast, in Wei et al.’s^34^ study using radiomic features to construct machine learning models for predicting PCR in rectal cancer, the LR predictive model had a higher PPV than KNN and SVM machine learning predictive models. The considerable differences between KNN, SVM predictive models, and the LR predictive model may be influenced by the original data.

In this study, we divided the data from our center into a training cohort and a test cohort, while data from other hospitals were used as an external validation cohort. There were no statistically significant differences in the basic demographic data between the cohorts, and the performance evaluation metrics of each machine learning model were generally consistent across the cohorts, ensuring the reliability and stability of each predictive model. Of course, there are certain limitations to our study. Firstly, our validation datasets were all from the same region, lacking international datasets for external validation. Secondly, although the RF predictive model improved the PPV, there was a significant decrease in the PPV of the predictive models in the test and validation cohorts. Lastly, the sample size of this study was very modest compared to studies with large samples to construct prediction models. A bigger sample of LAGC patients would be required to confirm the findings of this investigation in the future. For constructing predictive models with unbalanced binary classification samples, further research and exploration are needed to improve the PPV of the models, whether by finding new predictive models or identifying new clinical or radiomic features.

## Conclusion

In constructing predictive models for LAGC omental metastasis using unbalanced binary classification samples, the LR-based model has the disadvantage of low PPV and high false-positive rates. Among the machine learning algorithms, the RF predictive model, compared to LR, SVM, and KNN models, demonstrates higher accuracy while improving the PPV of the predictive model and reducing the false-positive rate. This makes the application of the model in the clinical treatment decision-making process more practical.

### Supplementary Information

Supplementary Information 1.

Supplementary Information 2.

## Data Availability

All data supporting the findings of this study are available within the paper and its Supplementary Information.

## Abbreviations

LAGC: Locally advanced gastric cancer
CT: Computed tomography
SVM: Support vector machine
DT: Decision tree
RF: Random forest
KNN: K-nearest neighbors
LR: Logistic regression
AUC: Area under the curve
ROC: Receiver operating characteristic
PPV: Positive predictive value
NPV: Negative predictive value
LASSO: Least absolute shrinkage and selection operator
AIC: Akaike information criterion
ICC: Intra-class correlation coefficient
DIOM: Diagnostics image original mean
OSMDS: Original shape maximum 2D diameter slice
OSMD: Original shape maximum 3D diameter
OFK: Original first-order kurtosis
WLFK: Wavelet LH first order kurtosis
WHGL: Wavelet HLH Gldm large dependence high gray level emphasis
OOB: Out-of-bag
PCR: Pathological complete response
LARC: Locally advanced rectal cancer

## References

[1] Sung H, Ferlay J, Siegel RL (2021). Global cancer statistics 2020: GLOBOCAN estimates of incidence and mortality worldwide for 36 cancers in 185 countries. CA Cancer J. Clin..

[2] Kim ST, Cristescu R, Bass AJ (2018). Comprehensive molecular characterization of clinical responses to PD-1 inhibition in metastatic gastric cancer. Nat. Med..

[3] Li S, Yu W, Xie F (2023). Neoadjuvant therapy with immune checkpoint blockade, antiangiogenesis, and chemotherapy for locally advanced gastric cancer. Nat. Commun..

[4] Anderson E, LeVee A, Kim S (2021). A comparison of clinicopathologic outcomes across neoadjuvant and adjuvant treatment modalities in resectable gastric cancer. JAMA Netw. Open.

[5] Shitara K, Bang YJ, Iwasa S (2020). Trastuzumab deruxtecan in previously treated HER2-positive gastric cancer. N. Engl. J. Med..

[6] Scott LJ (2018). Apatinib: A review in advanced gastric cancer and other advanced cancers. Drugs.

[7] Ricci AD, Rizzo A, Brandi G (2021). DNA damage response alterations in gastric cancer: Knocking down a new wall. Future Oncol..

[8] Guven DC, Sahin TK, Erul E (2022). The association between albumin levels and survival in patients treated with immune checkpoint inhibitors: A systematic review and meta-analysis. Front. Mol. Biosci..

[9] Rizzo A, Mollica V, Ricci AD (2020). Third- and later-line treatment in advanced or metastatic gastric cancer: A systematic review and meta-analysis. Future Oncol..

[10] Viscardi G, Tralongo AC, Massari F (2022). Comparative assessment of early mortality risk upon immune checkpoint inhibitors alone or in combination with other agents across solid malignancies: A systematic review and meta-analysis. Eur. J. Cancer.

[11] Santoni M, Rizzo A, Mollica V (2022). The impact of gender on the efficacy of immune checkpoint inhibitors in cancer patients: The MOUSEION-01 study. Crit. Rev. Oncol. Hematol..

[12] Huang C, Liu H, Hu Y (2022). Laparoscopic vs open distal gastrectomy for locally advanced gastric cancer: Five-year outcomes from the CLASS-01 randomized clinical trial. JAMA Surg..

[13] Hyung WJ, Yang HK, Park YK (2020). Long-term outcomes of laparoscopic distal gastrectomy for locally advanced gastric cancer: The KLASS-02-RCT randomized clinical trial. J. Clin. Oncol. Off. J. Am. Soc. Clin. Oncol..

[14] Chen M, He FQ, Liao MS, Yang C, Chen XD (2021). Gastrectomy with omentum preservation versus gastrectomy with omentectomy for locally advanced gastric cancer: A systematic review and meta-analysis. Int. J. Surg..

[15] Lin HW, Loh EW, Shen SC, Tam KW (2022). Gastrectomy with or without omentectomy for gastric cancer: A systematic review and meta-analysis. Surgery.

[16] Lambin P, Leijenaar RTH, Deist TM (2017). Radiomics: The bridge between medical imaging and personalized medicine. Nat. Rev. Clin. Oncol..

[17] Wang Y, Liu W, Yu Y (2020). CT radiomics nomogram for the preoperative prediction of lymph node metastasis in gastric cancer. Eur. Radiol..

[18] Cui Y, Zhang J, Li Z (2022). A CT-based deep learning radiomics nomogram for predicting the response to neoadjuvant chemotherapy in patients with locally advanced gastric cancer: A multicenter cohort study. EClinicalMedicine.

[19] Huang L, Feng B, Li Y (2021). Computed tomography-based radiomics nomogram: Potential to predict local recurrence of gastric cancer after radical resection. Front. Oncol..

[20] Sun R, Limkin EJ, Vakalopoulou M (2018). A radiomics approach to assess tumour-infiltrating CD8 cells and response to anti-PD-1 or anti-PD-L1 immunotherapy: An imaging biomarker, retrospective multicohort study. Lancet Oncol..

[21] Jiang Y, Wang W, Chen C (2019). Radiomics signature on computed tomography imaging: Association with lymph node metastasis in patients with gastric cancer. Front. Oncol..

[22] Jiang Y, Xie J, Han Z (2018). Immunomarker support vector machine classifier for prediction of gastric cancer survival and adjuvant chemotherapeutic benefit. Clin. Cancer Res. Off. J. Am. Assoc. Cancer Res..

[23] Zhao L, Han W, Niu P (2023). Using nomogram, decision tree, and deep learning models to predict lymph node metastasis in patients with early gastric cancer: A multi-cohort study. Am. J. Cancer Res..

[24] Tian S, Yu R, Zhou F (2023). Prediction of HER2 status via random forest in 3257 Chinese patients with gastric cancer. Clin. Exp. Med..

[25] Fan Z, Guo Y, Gu X, Huang R, Miao W (2022). Development and validation of an artificial neural network model for non-invasive gastric cancer screening and diagnosis. Sci. Rep..

[26] Li C, Zhang S, Zhang H (2012). Using the K-nearest neighbor algorithm for the classification of lymph node metastasis in gastric cancer. Comput. Math. Methods Med..

[27] Huang CM, Huang MY, Huang CW (2020). Machine learning for predicting pathological complete response in patients with locally advanced rectal cancer after neoadjuvant chemoradiotherapy. Sci. Rep..

[28] Wu A, Wu C, Zeng Q (2023). Development and validation of a CT radiomics and clinical feature model to predict omental metastases for locally advanced gastric cancer. Sci. Rep..

[29] Peng J, Wang W, Jin H (2023). Develop and validate a radiomics space-time model to predict the pathological complete response in patients undergoing neoadjuvant treatment of rectal cancer: An artificial intelligence model study based on machine learning. BMC Cancer.

[30] Jongerius EJ, Boerma D, Seldenrijk KA (2016). Role of omentectomy as part of radical surgery for gastric cancer. Br. J. Surg..

[31] Murakami H, Yamada T, Taguri M (2021). Short-term outcomes from a randomized screening phase II non-inferiority trial comparing omentectomy and omentum preservation for locally advanced gastric cancer: The TOP-G trial. World J. Surg..

[32] Ri M, Nunobe S, Honda M (2020). Gastrectomy with or without omentectomy for cT3-4 gastric cancer: A multicentre cohort study. Br. J. Surg..

[33] Huh JW, Kim HR, Kim YJ (2013). Clinical prediction of pathological complete response after preoperative chemoradiotherapy for rectal cancer. Dis. Colon Rectum.

[34] Wei Q, Chen Z, Tang Y (2023). External validation and comparison of MR-based radiomics models for predicting pathological complete response in locally advanced rectal cancer: A two-centre, multi-vendor study. Eur. Radiol..

[35] Núñez E, Steyerberg EW, Núñez J (2011). Regression modeling strategies. Rev. Esp. Cardiol..

[36] Strobl C, Malley J, Tutz G (2009). An introduction to recursive partitioning: Rationale, application, and characteristics of classification and regression trees, bagging, and random forests. Psychol. Methods.

[37] Ho DSW, Schierding W, Wake M, Saffery R, O'Sullivan J (2019). Machine learning SNP based prediction for precision medicine. Front. Genet..

[38] Sapir-Pichhadze R, Kaplan B (2020). Seeing the forest for the trees: Random forest models for predicting survival in kidney transplant recipients. Transplantation.

[39] Lorenzo D, Ochoa M, Piulats JM (2018). Prognostic factors and decision tree for long-term survival in metastatic uveal melanoma. Cancer Res. Treat..

[40] Romero MP, Chang YM, Brunton LA (2020). Decision tree machine learning applied to bovine tuberculosis risk factors to aid disease control decision making. Prev. Vet. Med..

[41] Luo X, Wen X, Zhou M, Abusorrah A, Huang L (2022). Decision-tree-initialized dendritic neuron model for fast and accurate data classification. IEEE Trans. Neural Netw. Learn. Syst..

